# Uterine Transplantation for Absolute Uterine Factor Infertility: From Bench to Bedside

**DOI:** 10.7759/cureus.100471

**Published:** 2025-12-31

**Authors:** Jesus Alberto Sanson-Riofrio, Ismael Brito-Toledo, Angelica Morelia, Alvar J Vacio Olguin, David Rayas Ruiz, Maria del Rosario Garcia, Roberto D Robles, Soledad Ruiz-Matus, Patricia Goldstein, Manuel M Meraz

**Affiliations:** 1 Surgical Oncology, Mexican Institute of Social Security, Mexico City, MEX; 2 Colon and Rectal Tumors, Oncology Hospital, National Medical Center Century XXI, Mexico City, MEX; 3 Epidemiology, Hospital General de Zona No. 33, Mexican Institute of Social Security, Mexico City, MEX; 4 Surgical Oncology, Oncology Hospital, Centro Médico Nacional Siglo XXI, Mexico City, MEX; 5 Intensive Care, Mexican Institute of Social Security, Mexico City, MEX; 6 Physics, National Autonomus University of Mexico, Mexico City, MEX; 7 Physics, Facultad de Ciencias Universidad Nacional Autonoma de Mexico, Mexico City, MEX; 8 Postgraduate Studies and Research, Medicine, Escuela Superior de Medicina - Instituto Politécnico Nacional (ESM IPN), Mexico City, MEX

**Keywords:** absolute uterine factor infertility (aufi), assisted reproduction, pregnancy, reproductive medicine, uterus transplantation

## Abstract

Uterus transplantation (UTx) has emerged as a promising reproductive option for women with absolute uterine factor infertility, and this narrative review offers an integrated translational overview of its progression from experimental research to clinical practice based on a structured qualitative synthesis of the literature retrieved from PubMed/MEDLINE, Embase, and Scopus between 2000 and June 2025, including original studies, reviews, case reports, meta-analyses, and clinical guidelines while excluding grey literature, incomplete abstracts, and non-human studies. Without performing quantitative synthesis or meta-analysis, the review highlights key historical milestones, advances in microsurgical technique and vascular preservation, evolving immunosuppressive strategies, reported graft function, reproductive and neonatal outcomes, and ethical considerations related to donor safety and equitable access. The review also notes that more than 90 uterine transplant procedures worldwide have led to over 50 reported live births, thereby demonstrating growing clinical reliability, while underscoring persistent challenges, such as graft rejection, infectious risk, and the long-term effects of immunosuppression, as the field continues to mature toward broader clinical applicability.

## Introduction and background

Human reproduction is a complex and tightly regulated biological process essential for species preservation. Despite its central role, human reproductive efficiency is relatively low, with an estimated probability of conception of only 30-35% per fertile cycle under optimal conditions. Multiple biological, environmental, and lifestyle factors may interfere with this process, leading to infertility - classically defined as the inability to achieve pregnancy after 12 months of unprotected intercourse [1]. Current global estimates indicate that infertility affects nearly one in six reproductive-age couples, generating not only medical challenges but also profound emotional, social, and economic consequences for those affected [1,2].

Among female causes of infertility, absolute uterine factor infertility (AUFI) represents a distinct clinical entity characterized by the complete inability to achieve pregnancy due to congenital uterine absence, surgical removal, or severe uterine dysfunction incompatible with implantation or gestation. AUFI accounts for approximately 3%-5% of women with infertility and has traditionally restricted reproductive options to surrogacy - often legally limited in many regions - or adoption, neither of which enables a genetically related pregnancy or the personal experience of gestation [3].

In this context, uterus transplantation (UTx) has emerged as a transformative surgical and reproductive innovation. Conceptualized decades ago but clinically realized only in the 21st century, UTx is currently the only intervention capable of restoring fertility in women with AUFI. The procedure involves transplanting a functional uterus from either a living donor (LD) or a deceased donor meeting the brain-death criteria (DBD) [3,4]. Since the first successful human cases, UTx has evolved from isolated experimental attempts to structured clinical programs with reproducible outcomes, reflecting major advances in transplant immunology, microsurgical techniques, reproductive endocrinology, and multidisciplinary care [3,4].

Beyond its medical relevance, UTx carries significant psychosocial implications, offering women with AUFI the unprecedented opportunity to experience pregnancy, childbirth, and autonomous motherhood - outcomes unattainable through adoption or conventional assisted reproductive technologies. Against this background, this narrative review aims to provide an integrated overview of the development of UTx, tracing its evolution from experimental models to contemporary clinical practice, while summarizing surgical approaches, donor considerations, reproductive outcomes, immunologic challenges, and ethical implications. The review is organized to first address historical and experimental foundations, followed by clinical implementation and outcomes, and concluding with current challenges and future directions. The rapid global expansion of the field, supported by international collaboration and continued technical refinement, highlights the potential of UTx to become an increasingly accessible therapeutic modality as evidence continues to grow.

History of UTx

The concept of replacing organs to restore physiological function has existed since antiquity, but true clinical UTx only became possible in the 20th century following advances in anesthesia, vascular surgery, and immunology [5,6]. Within this broader evolution, UTx emerged gradually, initially supported by experimental studies in animal models. Early milestones included the first documented uterine homotransplantation and autotransplantation in guinea pigs in 1918 by Hesselberg et al. [7], followed by uterine grafting experiments reported by Bykow and Yasnogorodsky in 1927 [8]. Subsequent work, such as swine autotransplantation by Butcher et al. and uterine segment UTx in small-animal models, contributed essential insights into reproductive physiology and vascular anastomosis [9,10]. By the 1960s, Eraslan et al. demonstrated successful uterine autotransplantation in dogs, marking a significant technical advance [11]. In parallel, the introduction of modern immunosuppressive therapies during the 1960s and 1970s markedly improved graft survival, laying the groundwork for later human applications.

The first human UTx attempt was reported in 2000 in Saudi Arabia using an LD, although the graft was removed on postoperative day 99 due to vascular thrombosis [12]. A decade later, the first deceased donor (DD) UTx was performed in Turkey, eventually resulting in a live birth after several early miscarriages, demonstrating the feasibility of DD programs [13]. A major breakthrough occurred in 2014 in Sweden, when Brännström et al. achieved the first live birth following LD-UTx, establishing the procedure as a viable treatment for AUFI [14]. This was followed in 2016 by the first successful DD-UTx live birth reported in Brazil, expanding global applicability and confirming the potential of DD procurement [15].

Since these milestones, multiple centers worldwide have implemented UTx programs, contributing to continual refinement of donor selection, surgical techniques, microsurgical approaches, and immunosuppressive management. Recent reviews highlight progressive improvements in graft survival, pregnancy rates, and neonatal outcomes, as well as significant advancements in minimally invasive techniques such as robotic-assisted donor hysterectomy [16,17]. As of June 2024, approximately 80 UTx procedures had been reported globally, resulting in 40 live births - 12 from DD donors and 28 from LD donors - reflecting the rapid maturation of the field [18-20]. The adoption of robotic-assisted donor surgery has further enhanced surgical precision, decreased morbidity, and improved operative times, particularly within LD-UTx programs [19,21].

Medical indications for UTx

Absolute uterine factor infertility - defined as the congenital or acquired absence of the uterus, or the presence of uterine dysfunction severe enough to prevent the establishment and maintenance of a pregnancy - represents the primary indication for UTx. The etiologies of this condition are diverse and encompass both anatomical and functional disorders, which are summarized in Table 1 [19,21].

**Table 1:** Clinical indications for uterus transplantation.

Indication	Description	Author/Year
Congenital uterine agenesis	Absence of the uterus from birth, as seen in Mayer-Rokitansky-Küster-Hauser syndrome.	Brännström et al., 2019 [19]
Uterine malformations	Structural anomalies that preclude gestation and cannot be corrected through standard surgical procedures.	Taran et al., 2019 [20]
Acquired uterine infertility	Loss of uterine function due to prior disease, trauma, or medical interventions.	Brännström et al., 2019 [19]
Previous hysterectomy	Women who have lost the uterus because of malignancy, postpartum hemorrhage, or severe uterine pathology.	Brännström et al., 2019 [19]
Functional uterine failure	Conditions such as extensive uterine fibrosis or severe intrauterine adhesions (Asherman syndrome) that impair implantation and pregnancy viability.	Brännström et al., 2019 [19]

In all these cases, the inability to achieve embryo implantation prevents a viable pregnancy, making UTx a reproductive alternative for these patients [19].

Donor Type

UTx may be performed using either an LD or a DD meeting DBD. DBD donors are individuals in whom all brain activity has irreversibly ceased, a determination established through neurological, functional, and imaging assessments. This condition permits the procurement of multiple organs in optimal physiological circumstances, thereby facilitating successful UTx [15,20,22].

The use of DBD donors provides several advantages over LD procurement. Most notably, it eliminates surgical risks for the donor, such as infection, wound dehiscence, hemorrhage, and inadvertent intraoperative injury. It also avoids postoperative complications associated with living-donor hysterectomy, including climacteric symptoms, dyspareunia, vaginal cuff dehiscence, gluteal claudication, hydronephrosis, urinary fistulas, and urinary incontinence [23-26].

Another critical advantage of DBD procurement is the ability to obtain longer vascular pedicles, which facilitates vascular anastomosis and enhances graft viability. Adequate pedicle length plays a key role in improving postoperative perfusion and increasing the likelihood of successful graft function [15,24-26]. This anatomical benefit directly contributes to more favorable clinical outcomes [27].

In contrast, UTx procedures involving LDs entail considerably longer surgical times - commonly 6-12 hours for the donor and 5-8 hours for the recipient. These prolonged operative durations extend warm and cold ischemia times, thereby compromising graft viability and increasing the risk of postoperative dysfunction [18,28-32].

The impact of ischemia on graft outcomes is well recognized. Prolonged warm or cold ischemia increases the risk of primary graft dysfunction or delayed graft function in solid-organ UTx [33-38]. Warm ischemia refers to the interval between arterial and venous occlusion and the initiation of cold perfusion. In contrast, cold ischemia refers to the period from ex vivo perfusion and storage at 4 °C until the completion of vascular anastomoses and reperfusion. Cold ischemia is particularly relevant in organs retrieved from DBD donors [39,40].

In human UTx, an upper limit for acceptable cold ischemia time has not been definitively established. Evidence suggests that the uterus may preserve its ultrastructural integrity for up to 24 hours and maintain contractile capacity for at least six hours under hypothermic storage. Reports from international UTx programs describe cold ischemia durations ranging from 1.3 to 6.6 hours, with successful graft and reproductive outcomes across this range [39-44].

During DBD procurement, hypothermic perfusion - achieved by infusing cold preservation solutions at 4 °C through the aorta and applying ice within the abdominal cavity - plays a central role in minimizing both warm ischemia and cellular injury. This strategy helps maintain tissue integrity and supports optimal graft condition before UTx [39].

Nevertheless, uterus retrieval from DBD donors is not without limitations. Potential challenges include variability in donor hemodynamic stability before procurement, the impact of prolonged intensive care support, exposure to vasopressors, and the risk of subclinical ischemia-reperfusion injury, all of which may influence graft quality. Additionally, logistical constraints, coordination among transplant teams, and legal or regulatory restrictions on reproductive organ donation may limit availability and standardization across centers. These factors underscore the importance of rigorous donor selection, optimized preservation protocols, and multidisciplinary coordination when utilizing DBD donors for UTx.

In summary, uterus donation from DBD donors represents the most feasible and ethically favorable approach for managing absolute uterine factor infertility. This modality avoids surgical morbidity in LDs while offering favorable graft viability and reproductive outcomes, thereby reinforcing its role as a promising therapeutic option for affected women [26,45-47].

Donor Evaluation Protocol in UTx

The selection of a uterus donor must adhere to strict medical criteria to ensure both graft viability and recipient safety [4,48,49]. LDs offer specific advantages, including improved preoperative optimization and shorter warm and cold ischemia times. The principal eligibility criteria for LD candidates are summarized in Table 2 [25,48].

**Table 2:** Selection criteria for living uterus donors. Abbreviations: HIV, human immunodeficiency virus; HBV, hepatitis B virus; HCV, hepatitis C virus; CMV, cytomegalovirus; EBV, Epstein–Barr virus; HSV, herpes simplex virus

Criterion	Definition	Author/Year
Age	Between 30 and 55 years	Johannesson et al., 2012 [25]
Obstetric history	At least one successful pregnancy, with a history of at least one term gestation	Carbonnel et al., 2024 [48]
Psychological evaluation	No evidence of personality or mood disorders; ensures voluntary, well-informed donation and understanding of surgical risks	Brännström et al., 2022 [49]
Health status	No chronic degenerative or rheumatologic disease; no infectious diseases (HIV, HBV, HCV, CMV, EBV, HSV); no gynecologic malignancy	Brännström et al., 2022 [49]
Assessment of uterine anatomy	Evaluation via Doppler and transvaginal ultrasound, and MR angiography to assess uterine vasculature	Carbonnel et al., 2024 [48]
Intraoperative assessment	Direct evaluation of uterine and vascular anatomy during procurement to confirm suitability	Johannesson et al., 2012 [25]

In the case of deceased DBDs, the selection criteria are modified due to the increased risk of prolonged ischemia. Donor age is generally restricted to 18-45 years, and the preferred cause of death is traumatic injury or primary neurological disease, while systemic infections must be avoided [26,50].

Accordingly, rigorous donor selection is essential to optimize uterine transplant outcomes and to minimize postoperative complications [37,43,51].

Recipient Evaluation Protocol in UTx

The evaluation of uterus transplant recipients follows principles similar to those applied in other solid-organ transplant protocols, with additional considerations unique to UTx. Among these, immunologic assessment and evaluation of ovarian reserve are particularly important, as they play a central role in optimizing transplant outcomes and reproductive success [22,52].

Immunologic compatibility is critical to reducing the risk of graft rejection. Human leukocyte antigen (HLA) typing is performed to determine the degree of compatibility between donor and recipient, as greater HLA matching is associated with lower rejection risk. Screening for anti-HLA antibodies is also essential, as their presence may trigger an immune-mediated response against the graft [53-55].

The immunologic workup additionally includes screening for viral infections such as cytomegalovirus (CMV), herpes simplex virus (HSV), and Epstein-Barr virus (EBV), all of which pose considerable risks to immunosuppressed patients. Immunosuppressive therapy - necessary to prevent graft rejection - requires strict monitoring to balance adequate immune control with the heightened susceptibility to infection [56,49].

Evaluation of Ovarian Reserve

Assessment of ovarian reserve is fundamental for determining the reproductive potential of the recipient and the overall feasibility of UTx. This evaluation relies on hormonal testing, ultrasonographic studies, and ovarian response assessments. The principal criteria are summarized in Table 3 [26,49,56].

**Table 3:** Evaluation of ovarian reserve in uterus transplant recipients. Abbreviations: AMH = Anti-Müllerian hormone; FSH = follicle-stimulating hormone

Diagnostic studies	Clinical Utility	Author/Year
Hormonal markers	Anti-Müllerian hormone (AMH), FSH, and estradiol used for a comprehensive assessment of ovarian function	Johannesson et al., 2023 [26]
Transvaginal ultrasound	Evaluates antral follicle count and ovarian morphology	Brännström et al., 2022 [49]
Controlled ovarian stimulation test	Determines ovarian responsiveness prior to the IVF cycle	Akbari et al., 2024 [56]
Ovarian function	Luteal-phase progesterone measurement guides reproductive decision-making	Brännström et al., 2022 [49]

The appropriate selection of recipients, grounded in a rigorous evaluation process, is essential for ensuring successful UTx and improving long-term outcomes [26,49,56].

Immune Response in UTx

Immunologic rejection in UTx is a complex process that may manifest as either acute or chronic rejection, depending on the interaction between the graft and the recipient’s immune system. Acute rejection typically occurs within the first weeks or months after UTx and is mediated by cytotoxic T lymphocytes that recognize the graft as foreign through major histocompatibility complex pathways, triggering cytokine-mediated inflammation and tissue injury [57].

In contrast, chronic rejection develops gradually and is characterized by progressive vascular changes within the transplanted uterus, ultimately leading to graft dysfunction and loss. Because this form of rejection may be clinically silent, routine surveillance through cervical biopsies and serological testing is critical for early detection and timely intervention [55].

To prevent rejection, an immunosuppressive regimen is implemented to balance graft preservation with maternal health and pregnancy viability. This strategy generally includes an induction phase using rapidly acting immunosuppressive agents, followed by a long-term maintenance protocol to support ongoing graft function. Adjuvant therapies may be used selectively to improve efficacy and reduce toxicity [57].

The UTx protocol at the Cleveland Clinic is based on a defined set of clinical and surgical criteria. Candidate selection focuses on non-smoking women aged 27-38 years, with a BMI between 21 and 25, and diagnoses such as Mayer-Rokitansky-Küster-Hauser (MRKH) syndrome, cervical cancer, or obstetric hemorrhage. Candidates must undergo IVF-ET within 12-18 months prior to UTx. Donors are typically healthy relatives. The protocol includes multidisciplinary evaluation and a surgical approach involving donor hysterectomy with vascular dissection, followed by arterial and venous anastomoses and vaginal anastomosis. Immunosuppressive therapy includes mycophenolate mofetil, prednisone, tacrolimus, azathioprine, and antithymocyte globulin [58,53,59,60].

Given the substantial risks associated with prolonged immunosuppression, post-cesarean hysterectomy is recommended once the birth of the first child has been achieved. This approach minimizes exposure to immunosuppressive agents and long-term complications. Thorough counseling is essential, and individualized immunosuppressive strategies are required [60-62].

Finally, clinical studies indicate that UTx success rates decline with subsequent pregnancies, increasing the risk of graft dysfunction and preterm delivery. Thus, UTx is considered a temporary intervention intended primarily for the birth of one child. Removal of the graft after a successful pregnancy reduces risks associated with long-term immunosuppression [63,64].

Methods

A narrative review was conducted to examine the historical development, surgical evolution, clinical outcomes, immunologic considerations, and ethical dimensions of UTx. The objective of this review was to provide a qualitative, translational synthesis of heterogeneous evidence spanning experimental research, early human experience, and contemporary clinical programs. Given the diversity of study designs, outcome definitions, and reporting standards in UTx, no quantitative synthesis, meta-analysis, or meta-regression was performed, as these approaches were not methodologically appropriate for the aims of this review.

A comprehensive literature search was performed in PubMed/MEDLINE, Embase, and Scopus, covering reports from the earliest descriptions of uterine autotransplantation in the early 20th century through June 2025, which was defined a priori as the search cutoff to ensure methodological consistency in a rapidly evolving field. Controlled vocabulary and free-text terms were used, including “uterus transplantation”, “uterine factor infertility”, “living donor uterus transplantation”, and “deceased donor uterus transplantation”, combined with Boolean operators to maximize retrieval. Filters were applied to identify human studies published in English or Spanish, including clinical trials, observational studies, case series, case reports, and systematic reviews [34,35].

Eligible studies included those describing historical or experimental milestones; surgical techniques in LDs or DDs; vascular and ischemia-related considerations; minimally invasive and robotic approaches; immunosuppression strategies; graft survival and function; reproductive and neonatal outcomes; and ethical, psychological, or social aspects of UTx. Exclusion criteria comprised non-human studies without direct clinical correlation, incomplete conference abstracts, grey literature, and reports lacking extractable clinical or methodological data [35,40].

Two reviewers independently screened titles and abstracts for relevance, followed by full-text assessment. Discrepancies were resolved by consensus. Study selection prioritized pioneering and foundational publications, as well as reports providing substantive clinical, reproductive, or ethical data from established programs, including the Swedish, Czech, Brazilian, and US DUETS experiences [12-15,21,27,38]. Older references were intentionally retained when they represented primary sources essential for accurately describing early milestones and avoiding secondary interpretation bias.

Data extraction was organized according to four predefined analytic domains: (1) historical development, including experimental autotransplantation models, insights into reproductive physiology, and early human procedures; (2) surgical evolution, with emphasis on donor procurement techniques, vascular dissection, strategies to mitigate warm and cold ischemia, and advances in laparoscopic and robotic donor hysterectomy [18,27,65,66]; (3) clinical and reproductive outcomes, including graft viability and function, rejection episodes, immunosuppressive regimens, pregnancy outcomes, obstetric complications, and neonatal results reported by major UTx cohorts [27,38,49]; and (4) ethical, psychological, and social considerations, encompassing donor risk-benefit assessment, informed consent processes, organ allocation considerations for DDs, and the psychosocial impact on both donors and recipients [35,40,46].

All included studies were critically appraised for relevance, internal coherence, and clarity of reporting. Given the narrative design, formal risk-of-bias assessment tools and Preferred Reporting Items for Systematic Reviews and Meta-Analyses (PRISMA) flow diagrams were not applied, as these are methodologically aligned with systematic reviews and quantitative syntheses. Findings were synthesized qualitatively and thematically to reflect the current state of evidence, ongoing challenges, and anticipated future directions in UTx.

Results

A total of 71 studies met the inclusion criteria and were incorporated into the qualitative synthesis, encompassing clinical trials, observational studies, case series, case reports, and systematic reviews. Collectively, these studies describe more than 90 UTx procedures worldwide, providing a comprehensive overview of the historical evolution of the field, surgical refinements, perioperative management, reproductive outcomes, and ethical considerations.

Early experimental investigations in animal models established the physiological feasibility of uterine grafting, defining principles of uterine vascularization, ischemia tolerance, and reproductive restoration that directly informed the first human procedures in the early 21st century [7-14].

Across clinical studies, surgical techniques evolved substantially. LD procedures progressed from open pelvic dissection to minimally invasive and robotic-assisted approaches, resulting in reduced donor morbidity in high-volume centers [18,27,65]. DD procurement expanded the donor pool and enabled longer vascular pedicles, although reported cold ischemia times ranged from approximately one to seven hours across programs, necessitating tailored preservation strategies to maintain graft viability [21,23,32-34].

Based on cases with sufficiently reported clinical follow-up, graft viability or functional survival was achieved in the majority of recipients, as evidenced by maintained perfusion, restored menstruation, or continued graft function during follow-up. Early graft loss was uncommon and occurred predominantly in initial pioneering experiences. Episodes of acute rejection were reported in a substantial proportion of recipients, most frequently within the first postoperative year, and were generally responsive to timely adjustment of immunosuppressive therapy [49,53].

Reproductive outcomes varied across programs. Among recipients with a functioning graft and reported reproductive follow-up, clinical pregnancy following embryo transfer was achieved in a significant proportion, and more than 50 live births have been reported globally to date. Pregnancies were frequently complicated by preterm delivery, with most births occurring between 32 and 37 weeks of gestation, and cesarean delivery was universally planned [14,21,27,38,49]. Neonatal outcomes were overall favorable, although a considerable proportion of infants required short-term neonatal intensive care due to prematurity [38,64].

Programmatic differences were observed in operative duration, ischemia thresholds, immunosuppressive regimens, and postoperative surveillance protocols, reflecting institutional experience and the evolving learning curve inherent to UTx implementation. These variations underscore the importance of standardized reporting and continued multicenter collaboration.

Ethical and psychosocial analyses consistently emphasized the need for rigorous informed consent, particularly for LDs exposed to major surgery without direct medical benefit, as well as careful consideration of organ allocation in deceased donation [35,40,42]. Psychological studies underscored the emotional burden experienced by both donors and recipients, supporting the integration of structured psychological assessment and longitudinal follow-up [46,59].

Overall, the collective results indicate that UTx is a feasible but highly specialized reproductive intervention, associated with meaningful graft survival and live birth outcomes, yet accompanied by substantial surgical, immunologic, and ethical challenges. Together, these findings delineate the current clinical landscape of UTx and inform its future development, as summarized in Table 4 [66,67].

**Table 4:** Reported uterus transplantation cases worldwide. Data represent published case reports, series, or program summaries. Countries with a single reported case are included for completeness but do not necessarily represent sustained national transplant programs. Abbreviations: LD = live donor; DBD = brain-death criteria

Country	Year(s)	Cases (n)	Approach	Reported Outcomes	Live Births	Author/Year (Ref)
Saudi Arabia	2000	1	LD → laparotomy	Graft loss at day 99	0	Fageeh et al., 2002 [12]
Turkey	2011/2021	1	DBD → laparotomy	Viable graft; miscarriages; later live birth	1	Ozkan et al., 2013 [13]
Sweden	2012	9	LD → laparotomy	7 Functioning grafts	9	Brännström et al., 2014 [14]
China	2015	1	LD robotic; R laparotomy	Successful graft	1	Brännström et al., 2019 [19]
China (multicenter series)	—	20	LD laparotomy/LD robotic/DBD	Not fully reported	—	Pecorino et al., 2024 [15]
United States (DUETS)	2016	20	Mixed (LD, DBD; laparotomy & robotic)	14 functioning grafts	14	Johannesson et al., 2023 [30]
Germany	2016	4	R → laparotomy	All grafts survived	2	Catsanos et al., 2020 [41]
Brazil	2016	1	DBD → laparotomy	Successful pregnancy	1	Nair et al., 2008 [42]
Lebanon	2018	1	LD → laparotomy	Graft functional	1	Järvholm et al., 2015 [46]
France	2019	1	LD robotic	Ongoing follow-up	—	Williams et al., 2016 [24]
Spain	2020	1	LD robotic	Successful graft	1	Johannesson et al., 2014 [68]
Italy	2020	1	DBD → laparotomy	Successful graft	1	Catsanos et al., 2020 [41]
Australia	2023	1	LD → laparotomy	Graft functional	—	Johannesson et al., 2023 [30]
Australia (case 2)	2023	1	LD → laparotomy	Graft functional	—	Johannesson et al., 2023 [30]

## Review

UTx represents a major advancement in the management of absolute uterine factor infertility, offering a reproductive option where none previously existed. It is the only intervention capable of enabling affected women to achieve gestation and childbirth with their own genetic offspring, fundamentally transforming the landscape of reproductive medicine [12-15,18,21,22,26,28,40,41,46,63]. Evidence from international programs has demonstrated both the technical feasibility of UTx and its capacity to achieve favorable reproductive outcomes in carefully selected candidates.

Despite its promise, UTx remains a complex, multistage intervention with significant surgical, medical, ethical, and psychosocial implications. The clinical course encompasses donor surgery, recipient UTx, postoperative stabilization, in vitro fertilization, pregnancy, delivery, and eventual graft removal. Each step requires meticulous monitoring to ensure graft viability, optimize maternal and fetal outcomes, and promptly address complications [18,27]. Immunosuppressive therapy remains one of the most challenging aspects of UTx: although essential for preventing rejection, it increases susceptibility to infection and carries metabolic and teratogenic risks during pregnancy. Because cumulative exposure heightens both maternal and fetal risk, most programs recommend limiting childbearing to a single live birth and performing hysterectomy thereafter to allow discontinuation of immunosuppression [31,49,50,53-55,64].

Twin pregnancies, though documented, appear to confer higher obstetric and graft-related risks and require intensified surveillance [4,14]. Successful pregnancies typically occur two to three years after UTx, although earlier conceptions have been reported once graft perfusion stabilizes [4,31,49]. These patterns underscore the importance of continuous assessment of vascular integrity, cervical biopsies for rejection monitoring, and embryo quality during assisted reproduction [27,38,49].

Ethical considerations remain central to the evolution of UTx. Living donors undergo extensive surgery without direct medical benefit, raising concerns about the risk-benefit balance and the adequacy of informed consent [35,42]. The use of deceased donors, while eliminating donor morbidity, introduces questions regarding organ allocation in systems designed primarily for life-saving UTx. Ensuring fairness, transparency, and ethical consistency in donor selection is therefore essential.

The limited availability of UTx is another significant challenge, influenced by the high cost of the procedure - estimated between $100,000 and $500,000 - and the need for highly specialized surgical and reproductive expertise [35,58]. As only a small number of centers worldwide have the resources and experience required, disparities in access persist, raising broader concerns about equity in reproductive care. Recipients, typically young and otherwise healthy women, face exposure to medications associated with infectious, metabolic, or oncologic risks, reinforcing the need for comprehensive counseling and individualized management plans [49,53,68].

Psychological dimensions are equally important. Donors and recipients may experience anxiety, uncertainty about surgical and reproductive outcomes, and emotional strain related to graft function and the possibility of rejection. Nonetheless, many recipients report significant improvements in emotional well-being and fulfillment after achieving pregnancy, underscoring the profound personal impact of UTx and the need for continuous psychological support throughout the process [46,59,68].

Taken together, UTx stands as a transformative yet inherently complex therapy. Its responsible implementation requires coordinated multidisciplinary collaboration, rigorous clinical monitoring, ethical vigilance, and structured psychosocial support. Continued refinement of surgical techniques - particularly minimally invasive and robotic donor procedures - along with advances in immunologic management and standardized follow-up protocols, has already contributed to improved safety and efficacy. As evidence grows and clinical practice expands, UTx is likely to become an increasingly feasible and ethically grounded reproductive option for women with absolute uterine factor infertility.

## Conclusions

UTx has advanced from an experimental intervention to a clinically feasible option for women with absolute uterine factor infertility, with growing evidence showing that, under careful candidate selection and coordinated multidisciplinary care, it can achieve stable graft function and successful pregnancies. Even with these encouraging results, UTx continues to involve considerable medical, surgical, and ethical challenges, particularly those related to immunosuppression, graft rejection, obstetric complications, and donor risk. Ongoing improvements in surgical technique, perioperative management, and immunologic protocols, together with responsible ethical oversight, will be essential for enhancing safety, standardizing clinical practice, and widening access to the procedure. In summary, UTx represents a meaningful reproductive alternative for selected women, and its future integration into routine clinical care will depend on sustained collaboration among clinical teams, researchers, and policymakers, as well as continued evaluation of long-term outcomes.
